# Facilitating a More Efficient Commercial Review Process for Pediatric Drugs and Biologics

**DOI:** 10.3390/diseases6010002

**Published:** 02017-12-22

**Authors:** Ryan D. Rykhus, Zachary V. Shepard, Alix Young, Hadley Frisby, Kailee A. Calder, Collin M. Coon, Justin A. Falk, Sydney R. McAndrews, Aspen Turner, Christina Chang, Johanna Michelsohn, Raegan Petch, Sarah M. Dieker, Benjamin H. Markworth, Kevin Alamo-Perez, Aaron J. Hosack, Jacob M. Berg, Christian Schmidt, Joachim Storsberg, Mark A. Brown

**Affiliations:** 1 School of Biomedical Engineering, Colorado State University, Fort Collins, CO 80523, USA; ryanrykhus@gmail.com (R.D.R.); ctchang@rams.colostate.edu (C.C.); bmarkworth@comcast.net (B.H.M.); 2 Department of Biomedical Sciences, Colorado State University, Fort Collins, CO 80523, USA; zshepard1216@gmail.com (Z.V.S.); alixyoung17@gmail.com (A.Y.); hfrisby@rams.colostate.edu (H.F.); kaileecalder@gmail.com (K.A.C.); aeturner@rams.colostate.edu (A.T.); jacob.berg@q.com (J.M.B.); 3 Department of Biology, Colorado State University, Fort Collins, CO 80523, USA; Collin.m.coon@gmail.com (C.M.C.); Sydney.r.mcandrews@gmail.com (S.R.M.); jmiche@rams.colostate.edu (J.M.); ahosack2017@gmail.com (A.J.H.); 4 Department of Mechanical Engineering, Colorado State University, Fort Collins, CO 80523, USA; Justin.falk98@gmail.com; 5 Department of Microbiology, Immunology, and Pathology, Colorado State University, Fort Collins, CO 80523, USA; rpetch@rams.colostate.edu; 6 Walter Scott College of Engineering, Colorado State University, Fort Collins, CO 80523, USA; dieksar17@yahoo.com; 7 Department of Electrical and Computer Engineering, Colorado State University, Fort Collins, CO 80523, USA; kevalamop777@gmail.com; 8 Department of Biomaterials and Healthcare, Fraunhofer-Institute for Applied Polymer Research (IAP), Division of Life Science and Bioprocesses, 14476 Potsdam-Golm, Germany; Christian.schmidt@iap.fraunhofer.de (C.S.); joachim.storsberg@iap.fraunhofer.de (J.S.); 9 Department of Clinical Sciences, Colorado State University, Fort Collins, CO 80523, USA; 10 Cell and Molecular Biology Program, Colorado State University, Fort Collins, CO 80523, USA; 11 Epidemiology Section, Colorado School of Public Health, Fort Collins, CO 80523, USA

**Keywords:** FDA, commercial drug approval, privatization, windfall tax

## Abstract

Over the past two decades, the biopharmaceutical industry has seen unprecedented expansion and innovation in concert with significant technological advancements. While the industry has experienced marked growth, the regulatory system in the United States still operates at a capacity much lower than the influx of new drug and biologic candidates. As a result, it has become standard for months or even years of waiting for commercial approval by the U.S. Food and Drug Administration. These regulatory delays have generated a system that stifles growth and innovation due to the exorbitant costs associated with awaiting approval from the nation’s sole regulatory agency. The recent re-emergence of diseases that impact pediatric demographics represents one particularly acute reason for developing a regulatory system that facilitates a more efficient commercial review process. Herein, we present a range of initiatives that could represent early steps toward alleviating the delays in approving life-saving therapeutics.

## 1. Introduction

The “evolving specter of pediatric disease” represents a global threat in public health [1]. This is manifest in the re-emergence of what were once thought to be well-controlled childhood diseases [2,3] and the fact that infectious diseases appear to be persisting among the leading causes of child mortality [4]. Even more troubling is the estimate that over 10,000 new cancer diagnoses will be made in U.S. children under the age of 15 this year, alone [5]. Despite major strides in the development of drugs and biologics targeting pediatric diseases, the limited capacity of the regulatory pathways for commercial approval of these products is exacerbating the existing bottleneck for the clinical management of preventable childhood deaths. 

As the submissions of new candidate drugs and biologics by industry sponsors continue to increase, the backlog for commercial reviews has become a growing thorn for FDA and sponsors, alike [6]. Rising frustrations among physicians and their patients demanding access to life-saving therapeutics has fueled a polarizing political climate calling for an overhaul of the commercial review process. Meanwhile, regulatory delays drive up costs of therapeutics, thereby creating a public relations nightmare for an industry that is already reeling from a tarnished reputation and a perceived inability to address patients’ needs. Estimates of the costs for developing drugs and biologics are rapidly approaching $3 billon [7,8]. In order to alleviate the mounting backlog, while maintaining high standards for safety and efficacy, a careful expansion of the regulatory approval process seems unavoidable. Unfortunately, in the past, increases in FDA funding have not led to commensurate increases in commercial approval times, indicating that attempts to directly expand federal regulation of the industry will likely exhibit only limited success [9]. 

Our ability to respond to the “evolving specter of pediatric disease” [1] requires appropriate steps to allow the biopharmaceutical industry to adequately address patients’ needs in a timely manner without compromising safety and/or diluting efficacy. The development of a new product approval framework that matches the rate of pharmacological innovation and mitigates costs due to regulatory delays will require broad input from a range of stakeholders. To initiate this process, we assembled an interdisciplinary panel to begin the search for innovative pathways for the appropriate streamlining of the approval of drugs and biologics. The initial outcomes of that panel are presented in the discussion, below. 

## 2. Discussion and Conclusions

**Figure 1:**
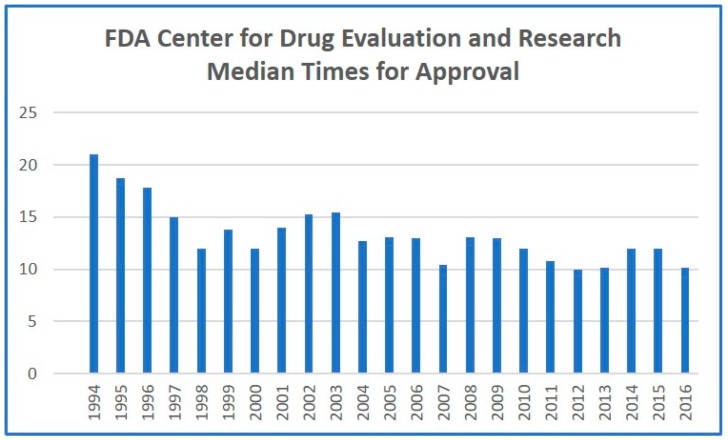
FDA center for drug evaluation and research median times (expressed in months) for approval since the year 1994.

Privatizing the FDA’s commercial approval process has been debated for over two decades [10]. We posit that total privatization of the process will expose the biopharmaceutical industry to daunting ethical issues that may compromise the integrity of the system. However, a partial privatization under FDA oversight could alleviate backlogs, while maintaining stringent regulatory standards. Privatized evaluation and approval companies could reduce product flow to FDA regulators by performing a similar service for an additional fee. This would allow pharmaceutical sponsors to pay for expedited approval, saving millions by bypassing regulatory delays. The precedent for this system was set by the Prescription Drug User Fee Act of 1992, which allowed the FDA to levy a fee to accelerate approval [9]. While this legislation has exhibited modest benefits in decreasing approval times (Figure 1), it has not had the desired effect of improving innovation rates or increasing the total number of products approved. However, private application of the same principle, coupled with expansion of the regulatory framework, should finally result in significant reduction in approval times, and, more critically, an increased number of drugs being allowed through the regulatory pipeline. More established pharmaceutical companies could justify the increased fees associated with privatized, accelerated approval, allowing the standard FDA approval process to focus on less-established pharmaceutical developers. This would have the added effect of accelerating traditional FDA approval, as the number of products arriving for standard approval would dramatically decrease. Competition among the privatized regulatory players would also incentivize greater efficiencies and fewer regulatory delays. 

To hold a new, partially privatized approval industry subject to the same standards as FDA evaluation, the FDA would need to be involved in directly overseeing and granting licensures to these companies, allowing them to operate in a similar scope to FDA regulators following evaluation for proper approval procedures. This initial time investment from the FDA, which, granted, would initially incur some additional costs on the Agency’s behalf, would be met with a system that, in addition to reducing product flow to the FDA, could also serve as additional oversight through the clinical trials process and to ensure ongoing adherence to current Good Manufacturing Practices (cGMP) throughout the approval process. This private system, in addition to leading to reduced expense for both the FDA and most pharmaceutical companies, would likely increase end-consumer safety beyond the present system, as has been the case in many instances of increased industry regulation [11].

Because regulatory companies would operate in a previously nonexistent branch of the industry, if the FDA is willing to provide the initial approval licensure, the economic incentive for initiating these private evaluation firms will be high. This will streamline the establishment of the system, providing a more rapid return on investment than direct funding increases to FDA have accomplished in the past. While private approval would potentially be subject to the same pitfalls that can befall any industry—corruption, nontransparent operations, and corner-cutting by individual companies that would pose additional risks for consumers—making the approval licensure subject to good standing with the FDA will have a similar regulatory effect on the approval industry that the presence of the FDA has on current product safety.

To establish such an approval industry, legislation would be necessary that would approve a pilot group to work with a team of FDA regulators to establish a framework of evaluation protocols, the blueprints for training evaluation staff in the private sector, and to thoroughly document the entirety of the FDA approval process such that it can be replicated in a private company. The early, small-scale tests of the concept should occur under direct FDA supervision to ensure that all procedures to ensure safety for the consumer are being followed and that the system will provide the predicted economic benefits that accompany accelerated approval. If all of this should prove successful, the framework will then exist for the expansion of pharmaceutical regulation and, potentially, the increase in innovation and product availability that the medical industry requires to continue growing.

Recognizing the daunting nature of privatizing the commercial approval process to the extent outlined above, our panel developed several initiating events that could ultimately precipitate a stepwise cascade to privatization. For example, the establishment of an Active Drug and Biologics Investigational Team (AuDIT), could consist of advisers who are trained to actively investigate pharmaceutical companies throughout each stage of the approval processes. From an efficiency perspective, this would have the effect of improving the rate of success among sponsors via real-time feedback in a system that emphasizes the formative nature of clearing the final hurdles of commercialization. Scaling from experimental-grade product to clinical-grade, and from pilot-scale manufacturing to commercial scale are common pitfalls which have resulted in the demise of more than a few products and sponsors. An AuDIT system, particularly one that includes in-house, FDA consultation, would better prepare sponsors to predict and respond to potential failures before they induce catastrophic financial costs and/or result in citations that haunt the sponsor throughout the regulatory path of the associated product. 

Other initiatives with the potential to improve the efficiency of the regulatory review process include concepts such as Independent Review Companies (IRCs) that could be tasked in a manner analogous to Contract Research Organizations (CROs) or a Universal Application Process. With IRCs, the FDA would have the option of assigning those aspects of the review process which comprise the greatest burden of time and resources to a third-party reviewer. With a Universal Application Process, each of the world’s leading biopharmaceutical regulatory agencies could collect Investigational New Drug and commercial approval applications in a uniform system through which each sponsor could select the regions in which they are seeking approval. There is, at least, modest precedent for such a system manifest in the translation of the FDA Guidances for Industry through the International Council for Harmonisation of Technical Requirements for Pharmaceuticals for Human Use (ICH). The Universal Application Process has the potential to improve efficiencies across regulatory agencies and sponsors alike, representing a significant savings for both. 

It is important to note that, in all of the various concepts, models, and initiatives developed and deliberated by our panel, the FDA remains an essential element for oversight. If the FDA is to remain central to the commercial review process, then funding for that agency must remain intact. This begs the question, where will the funds for new initiatives originate? Will this burden fall upon the shoulders of taxpayers? Will the biopharmaceutical industry be faced with these costs at the risk of stalling an industry that is poised for growth and innovation? Our panel endorses the concept of a biopharmaceutical windfall tax that mitigates these financial burdens by drawing funds from windfall profits at a rate that will collectively generate the required revenues, while representing only a small percentage of the profits for any individual product. To further prevent the stalling of successful sponsors, windfall taxes would be implemented only in exorbitant profit scenarios and only during a product’s period of exclusivity. 

Despite great innovations in the development of drugs and biologics for the clinical management of pediatric diseases, regulatory bottlenecks threaten to deny or delay children’s access to life-saving therapeutics. As human health researchers and as members of the biopharmaceutical community, we have a voice in this regulatory system, and an obligation to help improve it. We encourage the communities of pediatric health researchers, biopharmaceutical industry, and regulatory firms to come together and share ideas, concepts, and models toward facilitating a more efficient commercial review process for pediatric drugs and biologics.
